# Monitoring of white striping and wooden breast cases and impacts on quality of breast meat collected from commercial broilers (*Gallus gallus*)

**DOI:** 10.5713/ajas.18.0355

**Published:** 2018-08-27

**Authors:** Yuwares Malila, Juthawut U-chupaj, Yanee Srimarut, Premsak Chaiwiwattrakul, Tanaporn Uengwetwanit, Sopacha Arayamethakorn, Veerasak Punyapornwithaya, Chalutwan Sansamur, Catherine P. Kirschke, Liping Huang, Surapun Tepaamorndech, Massimiliano Petracci, Wanilada Rungrassamee, Wonnop Visessanguan

**Affiliations:** 1 Food Biotechnology Research Unit, National Center for Genetic Engineering and Biotechnology, Khlong Nueng, Khlong Luang, Pathum Thani 12120, Thailand; 2 Bio-sensing Technology Research Unit, National Center for Genetic Engineering and Biotechnology, Khlong Nueng, Khlong Luang, Pathum Thani 12120, Thailand; 3 Excellence Centre for Veterinary Public Health at Chiang Mai University, Muang, Chiang Mai, 50100, Thailand; 4 United States Department of Agriculture, Agricultural Research Service, Western Human Nutrition Research Center, Davis, CA 95616, USA; 5 Department of Agricultural and Food Sciences, Alma Mater Studiorum, University of Bologna, 47521 Cesena (FC), Italy

**Keywords:** White Striping, Wooden Breast, Commercial Broiler, Breast Meat, Meat Quality, Logistic Regression

## Abstract

**Objective:**

This study aimed at investigating white striping (WS) and wooden breast (WB) cases in breast meat collected from commercial broilers.

**Methods:**

A total of 183 breast samples were collected from male Ross 308 broilers slaughtered at the age of 6 weeks (n = 100) and 7 weeks (n = 83). The breasts were subjected to meat defect inspection, meat quality determination and histology evaluation.

**Results:**

Of 183, 4 breasts from 6-week-old broilers were classified as non-defective while the others exhibited the WS lesion. Among the 6-week-old birds, the defective samples from the medium size birds (carcass weight ≤2.5 kg) showed mild to moderate WS degree with no altered meat quality. Some of the breasts from the 6-week-old birds with carcass weight above 2.5 kg exhibited WB in accompanied with the WS condition. Besides of a reduction of protein content, increases in collagen matter and pH values in the defective samples (p<0.05), no other impaired quality indices were detected within this group. All 7-week-old broilers yielded carcasses weighing above 2.5 kg and showed abnormal characteristics with progressive severity. The breasts affected with severe WS and WB showed the greatest cook loss, hardness, springiness and chewiness (p<0.05). Development of WB induced significantly increased drip loss in the samples (p<0.05). Histology indicated necrotic events in the defective myofibers. Based on logistic regression, increasing percent breast weight by one unit enhanced the chance of WS and WB development with advanced severity by 50.9% and 61.0%, respectively. Delayed slaughter age from 6 to 7 weeks increased the likelihood of obtaining increased WS severity by 56.3%.

**Conclusion:**

Cases of WS and WB defects in Southeast Asia have been revealed. Despite few cases of the severe WS and WB, such abnormal conditions significantly impaired technological properties and nutritional quality of broiler breasts.

## INTRODUCTION

To meet growing consumer demand, broilers have been intensively selected for accelerating growth rate and enhanced muscle mass. Today, commercial broilers reach market weight within 5 to 8 weeks of age with enlarged breast muscle, the most valuable chicken part in broiler industry. The success in chicken production, however, has coincided with an increased incidence of quality abnormalities in chicken breast. The emerging white striping (WS), characterized by visual appearance of white lines parallel to muscle fiber on the surface of chicken breast meat, and wooden breast (WB), the meat exhibiting palpable hardness and muscle rigidity, are of great concern.

The causative factors of both WS and WB defects remain unclear. Several studies have suggested that such myopathies are likely the undesirable inherent side effect of the modern breeding selection [1–9]. As the breeding scheme has favored maximum growth rate and breast mass, vascularization has become limited within the muscle of the birds exhibiting such defective lesions [10,11]. In addition, inadequate oxygen supply and waste removal induce oxidative stress, necrosis and ultimately muscle damage [5,8,12].

The WS and WB occurrences have been extensively ad dressed in Western countries [1,3,4] over the past decade. In the early studies, the prevalence of WS chicken breast was reported to be approximately 10% depending on live weight of the chickens [3]. Within a few years, the incidence of WS has drastically increased, varying from 20% to as high as 96% [1,13]. The increased WS prevalence was also reported in birds experimentally fed with high-energy diets in comparison with low-energy diets [1,11]. In these studies, the defective breasts displayed negative changes in chemical composition, technological properties as well as consumer acceptance [2,3,14,15].

The occurrence of WS and WB defects in commercial broilers in Eastern and Southeast Asia has not been widely examined. One explanation could be the fact that most commercial broilers distributed in this region are generally slaughtered at the age of 6 weeks and sold with skin which may cover, if exists, any undesirable appearances. Only small percentage of broilers are reared longer, up to 7 weeks with an initial attempt to produce heavier broilers to be sold locally or exported to the Western countries in the forms of premium-grade whole carcasses or fresh cuts. Today, however, a growing number of heavy broilers are attained among birds that are reared within the normal period. Those rapidly grown birds are prone to the development of WS and WB which could adversely affect quality of the breast meat. The main objective of this study was to investigate WS and WB cases among commercial broilers slaughtered at 6 and 7 weeks of age at a commercial slaughterhouse in Thailand. Additional information regarding quality indices influenced by WS and WB defects was subsequently obtained. The association of the severity of WS and WB defects in the broiler breast meat with the slaughter age and breast yield was also analyzed using logistic regression.

## MATERIALS AND METHODS

### Sample collection

A total of 183 Ross 308 male broilers at 6 (100 birds) and 7 weeks (83 birds) of age were used in this study. All birds were reared under the uniform standard practices. Slaughtering process was operated by the company’s trained staff according to the Halal standard procedure. On the day of slaughter, the birds were fasted for 12 h and subsequently transported to the commercial slaughtering facility at Saraburi, Thailand. The birds were manually hung on shackles, electrically stunned using a water bath, and slaughtered by a manual neck cut. The birds were then bled for 3 min, scalded at 70°C for 2 min, and subsequently put in a rotary drum picker for 30 s. Immediately after de-feathering and feet removal, chicken carcasses were randomly collected from the conveying belt. Weight of each carcass was recorded and immediately advanced to evisceration. One side of the breasts was dissected from each eviscerated carcass, placed in a plastic bag and kept on ice while being transferred to a Food Biotechnology Laboratory lab, BIOTEC (Pathum Thani, Thailand) within 5 h after slaughtering. Upon arrival, the samples were stored at 4°C until quality defect inspection. On the other side of the breasts, the *pectoralis major* muscle was dissected from the cranial part of the breast along with muscle fiber orientation (5×5×5 mm) and preserved within 4% paraformaldehyde buffer while being transported back to the laboratory. These 20-min postmortem skeletal muscle specimens were collected for histological investigation.

Based on the carcass weights (the weight of the birds with out feathers and feet), the birds were graded into “medium” or “heavy” groups using the cut-off value of 2.5 kg (medium, weight ≤2.5 kg; heavy, weight >2.5 kg) according to the local poultry industry.

### Classification of white striping and wooden breast

**Figure 1:**
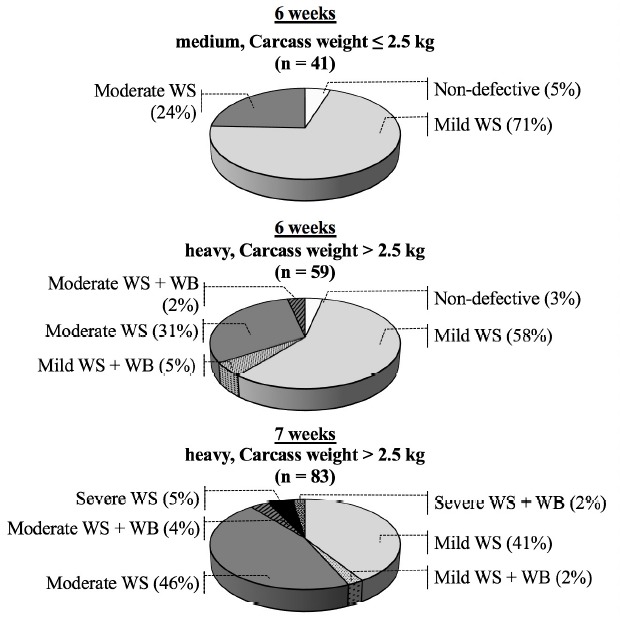
Cases of broiler breasts affected by white striping (WS) and wooden breast (WB) conditions. A total of 183 chicken carcasses were collected from commercial broilers (Ross 308) at the age of 6 weeks or 7 weeks and graded as “medium” and “heavy” based on the carcass weight (medium, weight ≤2.5 kg; heavy, weight >2.5 kg). No samples were fit within the category of “7 weeks, medium”.

After 24 h postmortem, each breast was individually weighed and subsequently inspected for WS and WB (Figure 1) using the criteria previously described with some modifications [4,14]. Briefly, the sorting was conducted by one trained staff throughout the investigation to minimize any variations. Samples exhibiting defects other than WS or WB, such as pale, soft, and exudative (PSE), were eliminated from this study. The WS severity was classified into four levels based on numbers and thickness of the white striation on the surface of the breast. Those categories include “non-WS” (no white striation found on the meat surface), “mild WS” (1 to 40 white lines with the thickness of ≤0.5 mm), “moderate WS” (more than 40 white lines or 1 to 5 line[s] with the thickness of 1.0 mm to 1.9 mm), and “severe WS” (more than 5 lines with the thickness of 1.0 mm to 1.9 mm or at least 1 line with thickness ≥2.0 mm). For WB, each breast fillet was classified as “WB” if there were substantial hardness and rigidity developing in the samples [4]. Immediately after inspection for WS and WB defects, the samples were subjected to pH measurements and preparation for further analyses.

### Meat quality determination

Evaluation of meat pH was conducted by inserting a spear-shape glass pH-probe (Mettler-Toledo Seven Easy, Mettler-Toledo, Inc., Greifensee, Switzerland) into three assigned positions (cranial, medial and caudal) of each meat sample. The cranial and caudal parts of the meat were then removed, thoroughly chopped and stored at −20°C until chemical composition analyses. The remaining portion, approximately 120 to 180 g, was used for determination of technological properties.

#### Chemical composition analysis

Moisture, protein and ash contents of the samples were determined following the recommended methods of AOAC [16]. Crude fat was extracted using a modified chloroform/methanol procedure described by Bligh and Dyer [17]. In brief, 10 g of chopped raw meat were homogenized for 2 min on ice in 80 mL of a solvent mixture consisting of 1 volume of chloroform, 2 volumes of methanol and 1 volume of distilled water. Twenty milliliters of chloroform were subsequently added into the homogenate and homogenized for 1 min. The homogenate was then mixed with 10 mL of distilled for 30 s before centrifugation at 3,000× g, 4°C for 15 min. The supernatant was then transferred into a separating flask and let stand for 1 h. The organic phase was filtered through a filter paper containing 2 g of anhydrous sodium sulfate into a pre-weighed dry round-bottom flask. The solvent was removed by evaporating at 65°C using a rotary evaporator. The crude fat was dried at 70°C for 4 h to remove any residual solvent and re-weighed afterwards. The fat to protein ratio was expressed in percentage. To estimate total collagen content, chopped raw breast meat was hydrolyzed in 10 volumes of 6 N HCl at 110°C for 24 h. After neutralization, hydroxyproline in the hydrolysate was determined according to the spectrophotometric method of Bergman and Loxley [18]. Hydroxyproline was converted into collagen by using conversion factor of 7.25 and expressed in milligram per gram meat sample. Percentage of collagen relative to crude proteins was also calculated. All measurements were carried out in duplicates.

#### Technological properties

Water holding capacity, in terms of drip loss and cook loss, as well as texture of cooked meat were assessed on each sample. Drip loss and cook loss were determined sequentially using the same sample. Following the trimming off the cranial and caudal parts, the meat was weighed and individually packed in a plastic bag and hung at 4°C for 24 h. The meat was re-weighed. Drip loss was expressed as the percentage of the weight loss due to gravitational force after 24-h hanging relative to the initial weight. Afterwards, the meat was vacuum-packed in a polyethylene plastic bag and cooked at 95°C by water immersion method. Internal temperature in the thickest portion of the meat was monitored throughout the cooking using a thermocouple. When the core temperature reached to 80°C, the meat was then cooled in an iced water until the temperature declined to below 15°C. The cooked meat was stored at 4°C for at least 2 h before weighing. Cook loss was represented as the percentage of the weight loss owing to cooking. Texture of the cooked samples was evaluated according to texture profile analysis using a TA-XTi texture analyzer (Stable Micro System, Godalming, UK) following the protocol described by U-chupaj et al [19]. Each breast was cut parallel to muscle fiber alignment to minimize the variation from muscle fiber direction into three cubes (10×10×10 mm). All textural parameters were automatically calculated and reported by the Exponent software (Stable Micro Systems, Goldalming, UK).

### Histological evaluation

The 20-min postmortem muscle samples collected for histological analysis were obtained from 6-week-old birds. The same region of the breast muscle was used to minimize variations due to differences in anatomical location. Upon arrival the laboratory, the tissues were fixed within 4% paraformaldehyde for 24 h at room temperature and subsequently embedded in paraffin. The specimens were cut into 5-μm transverse sections, stained with hematoxylin and eosin (H&E staining) and viewed under a bright-field microscope at 20× magnification.

### Statistical analysis

The statistical analysis was performed using the R package version 3.2.1. The dataset was firstly subjected to normality and variance equality testing using Shapiro-Wilk Normality Test and Bartlett Test, respectively. Mean difference among the treatments was analyzed using analysis of variance (ANOVA). The Duncan’s new multiple range test was used for post hoc multiple comparisons. The dataset that failed to follow normality and homogeneity of variance assumptions, were transformed using function *varIdent* from library *nlme* of R package to account for the difference in variance prior to ANOVA and multiple range test. The significant level for all statistical analyses was set as α = 0.05.

To assess the meat quality aberration in association with slaughter age and breast yield, two logistic regression models were constructed. The logistic regression was chosen herein because the regression responses, i.e. defective levels, were categorical variables. The general model is as follow.

$$\text{logit }[P\;(Y\leq j|{\text{X}}_{1},{\text{X}}_{2},\ldots ,{\text{X}}_{\text{k}})]=\alpha_{\text{j}}+\beta_{\text{j}1}{\text{X}}_{1}+\beta_{\text{j}2}{\text{X}}_{2}+\ldots +\beta_{\text{jk}}{\text{X}}_{\text{k}}$$

Where *P* (*Y*≤*j* | X_1_,X_2_,…,X_k_) is the probability of being at or below category *j*, given a set of X independent variables, α_j_ is an intercept, X_1_, X_2_,…X_k_ denote the independent variables and β_ik_ is the logistic coefficient for the *j*th category and *k*th independent variable. In this study, slaughter age and the percent breast weight were assigned as independent variables in all regression models. The model 1 described the effects of slaughter age and breast yield on WS severity following ordinal logistic regression (OLR). Severity of WS defect was defined as the ordinal response with non-WS = 1, mild WS = 2, moderate WS = 3 and severe WS = 4. With R, a function *polr* from *MASS* package was used for the analysis. The model 2 was designed as a binomial logit model focusing on the association between the independent variables and development of WB (non-WB = 1, WB = 2) and tested using generalized linear model function *glm*. The results from the logistic regression analysis were reported as the odds ratio. The odds ratio >1 indicates an increased chance whereas <1 denotes a decreased chance of a dependent category as a result of an increase in the continuous independent variable by one unit.

## RESULTS

### Cases of white striping and wooden breast abnormalities

The cases of the WS and WB abnormalities observed in this investigation are summarized in Figure 1. Of 183, 179 breasts, accounted for 97.8% of the total samples, exhibited the WS defect at various severity levels. Numbers of the samples categorized as mild WS, moderate WS and severe WS were 102 (55.7%), 71 (39.0%), and 6 (3.3%) samples, respectively. Besides, the presence of WB breasts was 6.6% of the total samples. Among the broilers slaughtered at 6 weeks of age, a total of four non-defective breasts, two among the medium and the other two among the heavy birds, were identified. No samples classified as severe were detected among those 6-week-old (6w) medium birds. Some of the 6w heavy birds, however, exhibited the WB condition in concert with the WS lesions. By extending slaughter age from 6 to 7 weeks, all birds, yielding carcasses weight above 2.5 kg, developed abnormal characteristics with progressive severity.

### Impact of white striping and wooden breast abnormality on breast meat quality

Table 1 reveals effects of WS and WB on the carcass traits and chemical compositions as well as technological properties of the breast samples. In addition to the defects, age and carcass weight were incorporated into this initial analysis to identify interactions among the two factors. Significant effects of the WS severity on the breast weight and yield, protein content, the ratio of fat to protein content, and cooked meat texture were observed (p<0.05). The meat developing WB exhibited aberrant meat quality in terms of breast yield, moisture, protein, ash content and water holding capacity of the breast samples (p<0.05). As expected, the two main effects, age and carcass weight, influenced broiler quality indices. Significant interactions were observed (p<0.05) between WS×WB, age×WS, and age×WB in some parameters. Interestingly, no interactions between carcass weight and other factors or among three or four factors were detected (p≥0.05).

For the carcass traits influenced by the WS and WB defects (Table 2), the birds slaughtered at 6 weeks of age exhibited significant increases in breast weight and yield as degree of WS and WB elevated (p<0.05). Although no statistical differences in the carcass traits were detected in the group of 7-week-old (7w) broilers (p≥0.05), all defective breasts obtained from such group tended to exhibit a greater breast proportion than those showing no defects. In this study, average breast yield of the non-defective meat was approximately at 14.7% whereas the defective breasts showed average breast yield ranged from 16.4% to 20.5%.

Effects of WS and WB on proximate composition (Table 3) and technological properties (Table 4) of the breast samples were more pronounced in the heavy broilers. Among the 6w heavy birds, superior moisture content was presented in the breasts classified as mild WS+WB (p<0.05). Protein content was significantly reduced in the defective samples compared to the non-WS samples (p<0.05). The WS severity, as the main effect in factorial model, significantly influenced fat to protein ratio. Once breaking down the broilers according to age and carcass weight, no significant differences in fat to protein ratio were observed among the defect degree (p≥0.05). For ash, no significant difference was found between non-WS and WS samples (p≥0.05) but the ash content significantly declined when the WS defect was accompanied with the presence of WB defect (p<0.05). Collagen content and the collagen to protein ratio were comparable between the non-defective and those of mild WS, mild WS+WB and moderate WS samples (p≥0.05) except for the moderate WS+WB breast samples which were significantly lower than those of the non-defective group (p<0.05). In addition, it was observed that the mild WS+WB and moderate WS+WB samples from 7w heavy broilers exhibited greater moisture but lower protein and ash than the others (p<0.05). Taken together, the current findings affirmed a reduced nutritional quality in terms of protein content in the WS and WB breasts.

For technological properties of the 6w heavy samples (Table 4), as WS severity advanced from mild to moderate degree, springiness of the cooked breast samples decreased but cohesiveness increased (p<0.05), suggesting the greater firmness of the latter samples. In the group of the 7w samples, development of WB induced greater drip loss in the breasts affected with mild and moderate WS (p<0.05). The breast samples with severe WS+WB showed the greatest degree of cook loss, hardness, springiness and chewiness (p<0.05), indicating inferior water holding capacity and tougher texture of the cooked WS+ WB meat.

### Histological analysis

**Figure 2:**
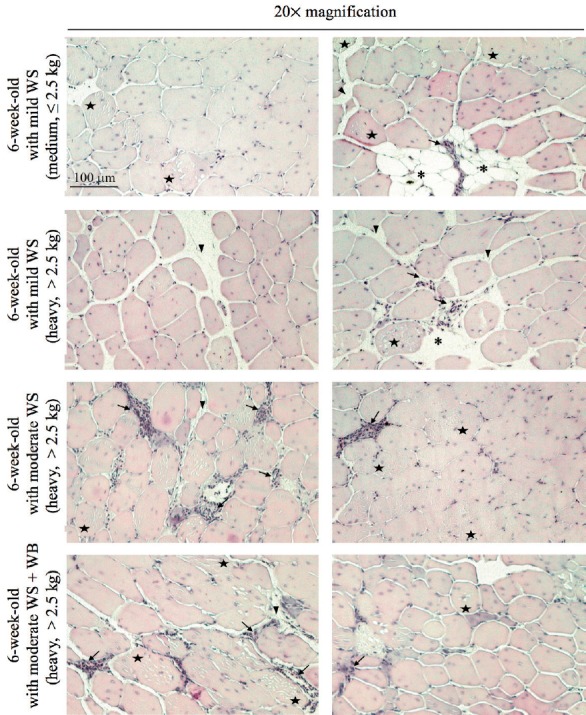
Histological images of the *pectoralis major* muscles depicted at 20× magnification. The breast muscle samples were collected from the broilers at the age of 6 weeks exhibiting various white striping (WS) severity with or without wooden breast (WB) condition. The broilers were graded as “medium” and “heavy” based on the carcass weight (medium, weight ≤2.5 kg; heavy, weight >2.5 kg). The fibers were eosin-stained and found as the polygonal structure. The nuclei were detected peripherally and internally in myofibers. Adipocytes (asterisks), macrophage deposition (arrows), and connective tissues (arrow heads) were found among the muscle fibers. Examples of necrotic fibers, observed as internal cell lesions, are marked with five-point stars. Scale bar = 100 μm for all images.

In this study, the muscle histology was evaluated in the *pectoralis major* muscles collected from 6w broilers exhibiting either mild WS, moderate WS or moderate accompanied with WB condition (Figure 2). Based on H&E staining, nuclear internalization and accumulation of macrophages were occasionally detected among the regular polygonal myofibers of the samples affected with mild WS defect. Deposition of adipocytes was observed. The thickened endomysial and perimysial connective tissues, particularly in the birds with carcass weight above 2.5 kg, may explain the increased total collagen content in the defective samples. As the severity condition progressively developed, the infiltration of macrophages became more apparent. The shape and diameter of the myofibers varied from small to large rounded, particularly in the muscle affected with moderate WS in concert with WB lesions. Additionally, necrotic fibers were commonly detected in the myofibers at all defective degrees. Together, the histological observations supported profound degenerative lesions and impaired regeneration of the myofibers in the WS and WB breast muscles.

### The likelihood of increasing defect severity associated with slaughter age and breast yield

In addition to examining the WS and WB defects, influence of slaughter age and percentage of breast weight on severity of WS or WB was analyzed using logistic regression. This allowed the estimation of the probability of obtaining WS and WB defective breast when the age and breast yield of the birds increased. According to the regression analyses (Table 5), breast yield was significantly associated with severity of both WS and WB defects (p<0.05). The odds ratios suggested that an increase in breast yield by one unit augmented the likelihood of the greater WS or WB condition by 50.9% (model 1) and 61.0% (model 2), respectively. The extended slaughter age was significantly associated only with the severity of WS (p≥0.05). The likelihood to obtain the WS defect with increased severity was at 56.3% when slaughter age was delayed from 6 to 7 weeks.

## DISCUSSION

Over the past decade, the increasing WS and WB prevalence in commercial broilers has triggered considerable concern among the poultry community. Although published reports regarding occurrence of WS and WB in Asian region are still limited, the incidence of both defects are noticeable in commercial slaughter plants. Based on the current study, 97.8% and 6.6% incidence of WS and WB, respectively, were detected among 183 collected male Ross 308 broilers, one of the two most produced commercial broiler lines in Southeast Asian region. The logistic regression models supported the association between WS and WB abnormalities and breast yield. The broilers with increased proportion of breast mass relative to the body size were more prone to develop WS and WB defects. This could explain the absence of severe WS and WB development in the medium 6w broilers which will be further discussed below. The relationship between increased breast yield and WS and WB defects agrees with previous reports [3,5,9,20,21]. By applying multinomial logistic regression, Kuttappan et al [1] demonstrated an increased WS degree with respect to a large cranial fillet thickness and heavy deboned carcass (p<0.05) regardless of chicken strains. A significant genetic correlation between WS defect with body weight and breast meat was also identified [7]. In the recent investigation of Kuttappan et al [21], linear and quadratic correlations between the severity degrees and breast weight were addressed in broilers slaughtered at 6 weeks of age. Furthermore, in the 7-week-old broilers, such relationship seemed to plateau as defective severity progressed. It must be kept in mind that the white line thickness cut-off value for severe WS in the previous investigations was 1.0 mm [14] of which was comparable to moderate WS level in this study. Recently, Sihvo et al [4] analyzed the development and morphology of the WB defect over the growth period in male Ross 508 broilers. In their study, the first WB cases were observed at the age of 18 days associated with mild macroscopic lesion focally developed in the caudal part of the breast. In the older age groups (35 to 42 days of age), the WB lesion appeared more severe accompanied with diffused characteristics.

Previous histological studies have consistently revealed myodegeneration within the defective muscle. The myopathic lesions have been shown to include multiple rounded muscle fibers with internalized nuclei [2,4,22] coupled with a diffuse thickening of endomysium and perimysium and loose collagen-rice connective tissue separating muscle fibers [6,8,22]. Despite overlapping microscopic characteristics, the WB breasts possess further degenerative severity with larger average muscle fiber diameter and cross-sectional area [8,23]. The muscle degeneration has been proposed to be a consequent phenomenon of which the hypertrophied fibers have outgrown the capacity of their supportive connective network [24]. In response to the damage, satellite cells are generally induced and begin to fuse with the existing myofibers or synthesize the new fibers, hence muscle repair. During muscle regeneration, sufficient vascularization is crucial for simultaneous formation of muscle and blood vessels. However, as capillary supply became limited in the muscle of birds with high breast yield [10,11], satellite cell-mediated muscle regeneration underwent impairment. Satellite cells might be converted into adipocytes accompanied with the replacement of the muscle fibers with collagen tissue [4,8,22], resulting in the phenotypic abnormalities in the defective meat. In the current study, the morphology of the myofibers from the 6w birds exhibiting different defect severity degrees was analyzed. Although the myopathic lesions observed herein were slightly milder, potentially due to the younger age of the birds, compared to those reported in previous studies [2,4,6,8,22], the findings supported the events of chronic muscle degeneration and aberrant regeneration regarding the WS and WB defects [2,4,8,22,24]. Histologically, the WS and WB conditions shared similar lesions. Still, the conclusion whether the two defects are the same etiology requires further investigation. The degenerative lesion has been linked with changes in chemical composition including increased moisture and fat coupled with reduced protein and ash in the abnormal meat [6,20]. The shift of proximate composition toward high fat and low protein proportion impaired nutritional profile of the abnormal chicken breast meat [15]. In addition, accumulation of collagen networks reduced protein quality [15] and contributed to cooked meat toughness in the defective breast [25].

The severe condition of the muscle disorders in the heavy 6w and 7w broilers observed herein well agreed with the aforementioned hypothesis regarding growth-induced myopathy. Those birds with increased breast yield exhibited progressive abnormal severity. The larger breast muscle mass likely induced the greater extent of muscle damage. In spite of no significant differences in fat content among the current samples, alterations of moisture, protein and ash were in agreement with previous studies. Total collagen of the samples obtained from the heavy 6w birds tended to increase in the WB samples. On the other hand, development of WS abnormality in the medium 6w birds followed similar trend where the birds with high breast yield exhibited elevated defect degree. However, breast and carcass weights of the medium birds were relatively lighter if compared with the rest of the collected samples. Mild condition of the degenerative muscles within such samples were anticipated thereby providing only minor impacts on phenotypic appearance and no effects on quality attributes.

An abnormally high ultimate pH in WS and WB breasts has been consistently evident among previous investigations [6,7,20,22,26] and has been also observed in the current study. The observation could not be clearly explained by the assumption of accumulated metabolic products owing to the inadequate muscular vascularization. If that was the case, lactic acid would be retained within the muscle cell, which lowered the ultimate pH of the resulting meat. Contrarily, the unusually high meat pH suggested an aberrant glucose metabolism within the defective breast muscle [7]. Dysregulation of lactic formation at molecular level either alone or in combination with limited glycogen stored in the defective muscle could be anticipated. The speculation has recently been supported by the decreases in transcripts [26], protein contents [27], and metabolites [12] relevant to glycolysis and gluconeogenesis in the myopathic muscles. It has been estimated that glycogen deposit in the non-defective muscles was 1.7-fold higher than that of the WB samples [12]. Although a comparison between glycogen content in normal and WS samples has not been reported yet, an increased protein abundance of lactate dehydrogenase isoform A in non-defective breasts for nearly 2-fold compared to that of the WS+WB samples [27] would manifest lactic acid formation within the WS breasts.

Distribution of water molecules in the WB defective breasts using a low-field nuclear magnetic resonance (NMR) has been previously assessed with an initial attempt to address the role of water in the WB meat rigidity [28]. The findings indicated decreases in the proportion of bound water and the water resided within the intra-myofibrillar space but a significant increase in extra-myofibrillar water fraction. Located between muscle bundles and sarcoplasm [29], the extra-myofibrillar water molecules loosely bound to the surrounding matrix in sarcoplasm by intermolecular forces, can be easily released from the cells by minor physical and mechanical forces. The migration of water molecules into extracellular space could be the explanation of the excessive drip and cook loss in the WB defective samples. The increased extra-myofibrillar water proportion in the defective breast has been proposed to be attributed by the histopathological myodegeneration [6,20]. As the muscle underwent damage, the water molecules embedded in the myofibrils and intracellular space between myofilaments have been released. This may be linked with the high moisture in the raw WB breast [6]. Employing the same NMR technique, Baldi et al [20] found no aberrant water migration within the WS affected meat which was in agreement with no effects of WS development on moisture losses observed herein and in previous study of Trocino et al [13]. In the studies of Petracci et al [3] and Soglia et al [22], however, an adverse effect of WS condition on water holding capacity of breast meat was detected. Decreases in myofibrillar protein quantity and functionality, potentially due to fibrosis and lipidosis, were suggested to be the responsive keys for the impairment [20,25]. The variation regarding water holding capacity of the defective meat could be the aspects of multiple factors, including broiler strain, slaughter age, sampling position and different cooking methods. Another possibility could be the heterogeneous myopathic lesions distributed throughout the meat [8,20].

Impacts of WS and WB defects on toughness of cooked breast meat, in terms of increasing shear force, have been addressed by some studies [3,26,30]. Corresponding to the current findings, Soglia et al [22] and Chatterjee et al [30] applied texture profile analysis to determine cooked meat texture and reported the greater degrees of hardness, springiness and chewiness of the WB and WS/WB breasts than those of non-defective meat. An increased toughness of cooked WS and WB meat has been hypothesized to be a consequence of the deposition of connective tissue, particularly collagen in the defective breasts [4,30]. An extensive fluid loss due to cooking plausibly intensified the toughness of the cooked meat as it reflected the shrinkage and densely packed muscle fibers after cooking [29]. However, altered textural characteristics of the defective meats were not consistent among reports as no difference in shear force between WS and normal cooked breast meat was also previously observed by Kuttappan et al [2], Alnahhas et al [7], and Trocino et al [13].

As addressed by Petracci et al [15], chicken breasts exhibiting severe WS (white lines thickness >1 mm) are usually downgraded and used in manufacturing meat processed products because of the blemish leading to decreased consumer acceptance. Only moderate WS breasts (white lines thickness <1 mm) are marketed in fresh retails. To our knowledge, a majority of either processors or consumers in this region are not aware of development of either WS or WB defects in the chicken breasts. In Thailand alone, numbers of consumers could not distinguish between non-defective and WS samples. Among those noticing the lines, they perceived the striation as normal appearance of the chicken breast. Thus, reduced visual attraction due to the WS defect may not be the major concern. However, because the defects have been neglected by the processors, the abnormality could silently be responsible for technological challenges, such as excessive water loss and inconsistent quality of further processed products. Additionally, nutritional quality of the moderate and severe WS breast may differ from consumer expectation.

## CONCLUSION

To the best of our knowledge, this study is among a few of the published documents regarding the WS and WB cases in Asian regions. While the origins of those defects are still under investigation, our findings were congruent with those of previous studies suggesting that the origin of the myopathies might be linked to the intensive broiler selection scheme for massive breast yield. Herein, the WS and WB defects in commercial male fast-growing broilers were evidently found among the birds with ages of 6 and 7 weeks. The groups of mild WS and moderate WS samples were the majority. Although the severe WS and WB were observed in a few cases, they showed an apparent technological impairment and diminishing nutritional quality that would adversely affect overall value of the broiler breast. To effectively mitigate the incidence of such defects, further comprehensive studies are crucial to elucidate causative factors and underlying mechanisms regarding both WS and WB defects.

## Figures and Tables

**Table 1:** Effects of slaughter age, carcass size and meat defects on properties of commercial broilers

Property	Main effect1)				Two-factor interaction2)		
	
	Defect		Age	Size	WS×WB	Age×WS	Age×WB

	WS	WB					
Carcass weight	ns	ns	***	***	ns	ns	ns
Breast weight	***	ns	***	***	ns	ns	ns
Breast yield	***	*	***	*	ns	ns	ns
Moisture	ns	***	**	*	ns	ns	*
Crude protein	**	***	***	*	**	ns	*
Crude fat	ns	ns	ns	ns	ns	ns	ns
Ash	ns	***	*	*	ns	ns	ns
Total collagen	ns	ns	*	ns	ns	ns	ns
Fat:protein ratio	*	ns	ns	ns	ns	ns	ns
Collagen:protein ratio	ns	ns	*	ns	ns	ns	ns
pH	**	ns	ns	ns	ns	ns	ns
Drip loss	ns	***	***	**	ns	ns	***
Cook loss	ns	*	***	*	ns	*	ns
Hardness	***	ns	**	ns	ns	ns	ns
Springiness	*	ns	ns	ns	ns	ns	*
Cohesiveness	*	ns	ns	ns	ns	*	ns
Chewiness	**	ns	***	**	ns	ns	ns

WS, white striping; WB, wooden breast.

1) Main effects consist of WS (non-WS, mild, moderate and severe), WB (non-WB and WB), age (6 wk and 7 wk) and carcass weight (weight ≤2.5 kg and weight >2.5 kg).

2) No interactions between carcass weight and other factors, among three-factor or four-factor levels were observed.

* p<0.05,

** p<0.01,

*** p<0.001,

ns, not significant (p≥0.05).

**Table 2:** Carcass traits of commercial broilers as affected by white striping and wooden breast defects1)

Age/carcass weight	Traits	Defect level						

		Non-WS	Mild WS		Moderate WS		Severe WS	
			
		Non-WB	Non-WB	WB	Non-WB	WB	Non-WB	WB
6 wk/≤2.5 kg	Sample size	2	29	0	10	0	0	0
	Carcass weight (kg)	2.3^b^±0.3	2.3^b^±0.1	nd	2.4^a^±0.2	nd	nd	nd
	Breast weight (g)	165.5^b^±7.4	193.1^a^±19.1	nd	208.2^a^±13.9	nd	nd	nd
	Breast yield (%)	14.8^b^±1.3	16.9^a^±1.5	nd	17.5^a^±1.4	nd	nd	nd
6 wk/>2.5 kg	Sample size	2	34	3	18	2	0	0
	Carcass weight (kg)	2.9±0.2	2.8±0.2	2.7±0.1	2.9±0.3	2.9±0.3	nd	nd
	Breast weight (g)	211.2^b^±32.8	228.5^b^±28.7	237.4^ab^±11.3	254.3^ab^±40.8	295.5^a^±24.8	nd	nd
	Breast yield (%)	14.6^c^±3.4	16.4^bc^±1.7	17.8^ab^±1.1	17.7^a^±1.8	20.4^a^±0.2	nd	nd
7 wk/>2.5 kg	Sample size	0	34	2	38	3	4	2
	Carcass weight (kg)	nd	3.2±0.2	3.3±0.1	3.3±0.2	3.4±0.3	3.4±0.4	3.1±0.2
	Breast weight (g)	nd	286.8±36.2	327.5±12.0	304.5±30.2	312.2±14.3	318.6±51.1	311.4±8.0
	Breast yield (%)	nd	17.8±1.6	19.7±0.23	18.3±1.5	18.6±1.3	19.0±1.3	19.9±1.9

WS, white striping; WB, wooden breast; nd, not detected, indicating that no samples were classified into such category.

1) Data are presented in mean±standard deviation.

a–c Different superscripts indicate statistical difference within each row (p<0.05).

**Table 3:** Chemical compositions of broiler breast meat as affected by white striping and wooden breast defects1)

Age/carcass weight	Composition	Defect level						

		Non-WS	Mild WS		Moderate WS		Severe WS	
			
		Non-WB	Non-WB	WB	Non-WB	WB	Non-WB	WB
6 wk/≤2.5 kg	Moisture (%)	75.4±0.7	74.9±0.7	nd	75.0±0.8	nd	nd	nd
	Crude protein (%)	22.2±0.1	22.3±1.0	nd	22.1±0.8	nd	nd	nd
	Crude fat (%)	1.3±0.2	1.3±0.4	nd	1.6±0.2	nd	nd	nd
	Ash (%)	1.21±0.06	1.21±0.07	nd	1.2±0.1	nd	nd	nd
	Total collagen (mg/g meat)	1.2^c^±0.3	2.6^b^±0.5	nd	3.2^a^±0.3	nd	nd	nd
	Fat:protein ratio (%)	5.9±0.8	6.8±1.6	nd	7.2±1.1	nd	nd	nd
	Collagen:protein ratio (%)	0.6^b^±0.1	1.2^a^±0.2	nd	1.4^a^±0.2	nd	nd	nd
6 wk/>2.5 kg	Moisture (%)	74.6^b^±0.3	75.0^b^±0.6	76.5^a^±1.5	75.0^b^±0.7	75.4^b^±1.14	nd	nd
	Crude protein (%)	23.9^a^±0.4	22.3^b^±0.7	20.3^c^±1.7	22.1^bc^±1.1	21.7^bc^±0.38	nd	nd
	Crude fat (%)	1.4±0.3	1.6±0.4	1.5±0.1	1.6±0.3	1.4±0.2	nd	nd
	Ash (%)	1.24^a^±0.02	1.19^ab^±0.08	1.08^bc^±0.23	1.18^ab^±0.06	0.97^c^±0.01	nd	nd
	Total collagen (mg/g meat)	1.6^b^±0.2	2.8^ab^±1.0	2.0^b^±0.1	2.7^ab^±0.7	4.2^a^±1.2	nd	nd
	Fat:protein ratio (%)	5.7±1.0	7.1±1.6	7.4±0.6	7.2±1.6	6.4±0.7	nd	nd
	Collagen:protein ratio (%)	0.7^b^±0.1	1.3^ab^±0.5	1.0^b^±0.01	1.2^ab^±0.3	1.9^a^±0.5	nd	nd
7 wk/>2.5 kg	Moisture (%)	nd	75.2^b^±1.1	78.4^a^±0.2	75.4^b^±1.0	77.5^a^±1.7	75.7^b^±2.5	76.9^ab^±0.1
	Crude protein (%)	nd	22.0^a^±0.9	18.6^c^±1.3	21.8^a^±1.0	18.7^b^±1.6	21.0^ab^±2.1	23.9^ab^±0.01
	Crude fat (%)	nd	1.5±0.3	1.4±0.1	1.7±0.5	1.6±0.6	2.0±1.3	1.6±0.2
	Ash (%)	nd	1.16^a^±0.08	1.03^b^±0.57	1.17^a^±0.67	1.06^b^±0.02	1.12^ab^±0.09	1.10^ab^±0.1
	Total collagen (mg/g meat)	nd	3.3±1.8	5.0±2.8	2.6±0.5	3.0±1.7	4.0±1.3	3.3±0.01
	Fat:protein ratio (%)	nd	6.9±1.4	7.8±0.9	7.7±2.1	8.3±2.5	9.8±6.9	7.5±1.2
	Collagen:protein ratio (%)	nd	1.5±0.9	2.7±1.7	1.2±0.3	1.6±1.1	2.0±0.8	1.5±0.1

WS, white striping; WB, wooden breast; nd, not detected, indicating that no samples were classified into such category.

1) Data are presented in mean±standard deviation.

a–c Different superscripts indicate statistical difference within each row (p<0.05).

**Table 4:** Technological properties of broiler breast meat affected by white striping (WS) and wooden breast (WB) defects1)

Age/carcass weight	Property	Defect level						

		Non-WS	Mild WS		Moderate WS		Severe WS	
			
		Non-WB	Non-WB	WB	Non-WB	WB	Non-WB	WB
6 wk/≤2.5 kg	pH	5.83±0.01	6.00±0.22	nd	5.94±0.13	nd	nd	nd
	Drip loss (%)	1.0±0.4	0.6±0.2	nd	0.7±0.2	nd	nd	nd
	Cook loss (%)	19.0±3.4	16.5±3.7	nd	16.4±2.5	nd	nd	nd
	Hardness (N)	25.0±0.04	26.1±5.5	nd	24.9±3.6	nd	nd	nd
	Springiness	0.63±0.01	0.65±0.03	nd	0.63±0.02	nd	nd	nd
	Cohesiveness	0.41±0.01	0.40±0.04	nd	0.42±0.07	nd	nd	nd
	Chewiness (N)	6.4±0.2	6.0±2.3	nd	6.6±1.3	nd	nd	nd
6 wk/>2.5 kg	pH	5.65^b^±0.08	6.00^a^±0.24	6.10^a^±0.25	5.98^a^±0.15	5.93^a^±0.19	nd	nd
	Drip loss (%)	1.1±0.3	0.7±0.4	0.8±0.2	0.7±0.3	0.9±0.5	nd	nd
	Cook loss (%)	17.2±1.6	16.5±4.9	16.0±3.5	12.8±4.1	10.5±4.0	nd	nd
	Hardness (N)	21.2±2.9	24.8±5.0	23.2±6.9	25.4±5.8	25.4±8.6	nd	nd
	Springiness	0.60^b^±0.04	0.64^a^±0.03	0.63^ab^±0.03	0.62^ab^±0.04	0.61^ab^±0.03	nd	nd
	Cohesiveness	0.42^ab^±0.01	0.42^b^±0.06	0.45^ab^±0.08	0.46^a^±0.06	0.47^a^±0.06	nd	nd
	Chewiness (N)	5.4±1.1	6.1±2.3	6.5±2.0	7.4±2.2	7.5±3.7	nd	nd
7 wk/>2.5 kg	pH	nd	5.95±0.13	6.06±0.23	5.97±0.10	6.03±0.12	6.07±0.07	5.98±0.02
	Drip loss (%)	nd	1.0^b^±0.5	2.1^a^±0.1	0.9^b^±0.5	2.0^a^±0.7	0.8^b^±0.2	1.1^ab^±0.0
	Cook loss (%)	nd	20.4^b^±3.3	20.3^b^±0.5	19.5^b^±4.0	24.5^ab^±9.0	22.1^b^±5.0	29.7^a^±1.4
	Hardness (N)	nd	27.4^b^±4.7	26.4^b^±8.5	28.3^b^±5.0	27.4^b^±7.9	30.9^b^±7.1	43.7^a^±5.1
	Springiness	nd	0.64^b^±0.05	0.67^ab^±0.04	0.64^b^±0.04	0.68^ab^±0.06	0.65^ab^±0.03	0.72^a^±0.01
	Cohesiveness	nd	0.43±0.07	0.50±0.01	0.42±0.07	0.41±0.10	0.37±0.04	0.42±0.02
	Chewiness (N)	nd	7.6^b^±1.5	9.0^b^±1.8	7.4^b^±1.5	7.7^b^±2.8	7.6^b^±2.5	13.5^a^±2.6

WS, white striping; WB, wooden breast; nd, not detected, indicating that no samples were classified into such category.

1) Data are presented in mean±standard deviation.

ab Different superscripts indicate statistical difference within each row (p<0.05).

**Table 5:** Coefficients, standard error and odds ratio for the variables included in the logistic regression models

Model	Predictor	Estimated coefficient	Standard error	Odds ratio (95% CI)	Residual deviance	AIC
Model 1:					377.93	393.93
White striping	Slaughter age (6 wk)	−0.829	0.321	0.437* (0.233, 0.820)	-	-
	Percent of breast weight	0.411	0.099	1.509*** (1.244, 1.830)	-	-
Model 2:					76.28	82.28
Wooden breast	Slaughter age (6 wk)	0.100	0.662	1.105 (0.302, 4.043)	-	-
	Percent of breast weight	0.476	0.190	1.610* (1.109, 2.335)	-	-

CI, confidence interval; AIC, akaike information criterion.

* p<0.05,

*** p<0.001.
